# Non-agglomerated silicon–organic nanoparticles and their nanocomplexes with oligonucleotides: synthesis and properties

**DOI:** 10.3762/bjnano.9.234

**Published:** 2018-09-21

**Authors:** Asya S Levina, Marina N Repkova, Nadezhda V Shikina, Zinfer R Ismagilov, Svetlana A Yashnik, Dmitrii V Semenov, Yulia I Savinovskaya, Natalia A Mazurkova, Inna A Pyshnaya, Valentina F Zarytova

**Affiliations:** 1 Novosibirsk State University, ul. Pirogova 2, Novosibirsk, 630090, Russia; 2 Institute of Chemical Biology and Fundamental Medicine, Siberian Branch of Russian Academy of Sciences, pr. Lavrent’eva 8, Novosibirsk, 630090, Russia; 3 Institute of Catalysis, Siberian Branch of Russian Academy of Sciences, pr. Lavrent’eva 5, Novosibirsk, 630090, Russia; 4 FBRI State Research Center of Virology and Biotechnology "Vector", Koltsovo, Novosibirsk region, 630559, Russia

**Keywords:** antiviral effect, non-agglomerated silicon–organic nanoparticles, penetration, Si–NH_2_·ODN nanocomplexes

## Abstract

The development of efficient and convenient systems for the delivery of nucleic-acid-based drugs into cells is an urgent task. А promising approach is the use of various nanoparticles. Silica nanoparticles can be used as vehicles to deliver nucleic acid fragments into cells. In this work, we developed a method for the synthesis of silicon–organic (Si–NH_2_) non-agglomerated nanoparticles by the hydrolysis of aminopropyltriethoxysilane (APTES). The resulting product forms a clear solution containing nanoparticles in the form of low molecular weight polymer chains with [─Si(OH)(C_3_H_6_NH_2_)O─] monomer units. Oligonucleotides (ODN) were conjugated to the prepared Si–NH_2_ nanoparticles using the electrostatic interaction between positively charged amino groups of nanoparticles and negatively charged internucleotide phosphate groups in oligonucleotides. The Si–NH_2_ nanoparticles and Si–NH_2_·ODN nanocomplexes were characterized by transmission electron microscopy, atomic force microscopy and IR and electron spectroscopy. The size and zeta potential values of the prepared nanoparticles and nanocomplexes were evaluated. Oligonucleotides in Si–NH_2_·ODN complexes retain their ability to form complementary duplexes. The Si–NH_2_^Flu^ nanoparticles and Si–NH_2_·ODN^Flu^ nanocomplexes were shown by fluorescence microscopy to penetrate into human cells. The Si–NH_2_^Flu^ nanoparticles predominantly accumulated in the cytoplasm whereas ODN^Flu^ complexes were predominantly detected in the cellular nuclei. The Si–NH_2_·ODN nanocomplexes demonstrated a high antisense activity against the influenza A virus in a cell culture at a concentration that was lower than their 50% toxic concentration by three orders of magnitude.

## Introduction

The development of efficient and convenient systems for the delivery of nucleic-acid-based drugs into cells is an urgent task. The solution to this problem would allow for the use of these drugs in practical medicine. Despite many efforts in this field, this problem cannot be considered as completely solved. А promising approach is the use of various nanoparticles as delivery vehicles. We have previously developed methods for immobilizing DNA fragments onto titanium dioxide nanoparticles with the formation of TiO_2_·PL–DNA nanocomposites [1–2]. Silica nanoparticles can also be used as vehicles to deliver nucleic acid fragments into cells [3–4].

SiO_2_ nanoparticles bearing amino groups on the surface were shown to bind plasmid DNA, allowing the nanoparticles to penetration into cells, and even nuclei, and to protect DNA against intracellular nucleases [5–6]. The prospect of using SiO_2_ nanoparticles as nonviral nanovectors to deliver plasmid DNA and their lower toxicity compared to the widely used transfection agent lipofectamine was shown in previous work [6]. It was demonstrated that SiO_2_ nanoparticles with a diameter of 20–77 nm can easily penetrate into cellular cytoplasm and nuclei [7]. Polylysine-modified nanoparticles appeared to be nontoxic upon oral administration in mice, which opens the possibility for their wide application [8]. Under physiological conditions, SiO_2_ nanoparticles are known to degrade to orthosilicic acid, Si(OH)_4_, which is found in almost all human tissues and effectively excreted through the urine [9]. Organically modified silica nanoparticles are known for their low toxicity and biocompatibility and can be used for targeted imaging and therapy [10–11].

The synthesis of amino-containing silica nanoparticles has been described in many works [3,6,12–15]. Most often, amino-containing silica nanoparticles are prepared by copolymerization during the hydrolysis of alkyl (or aryl)triethoxy(or methoxy) silanes in the presence of APTES [16–17]. A large series of works describes the synthesis of amino-containing silicon nanoparticles, so-called ORMOSIL, and their use for diagnostics and therapy [15,18]. Most often, these methods lead to the formation of nanoparticles with a diameter of 20–100 nm. Silica is particularly attractive as a functional, biocompatible, nontoxic, and inert material, which can be easily synthesized and modified [16–17].

In this work, we describe the synthesis of silicon-based nanoparticles by the hydrolysis of APTES alone, the preparation of oligonucleotide-containing nanocomplexes on their basis, and characterization of both nanoparticles and nanocomplexes. We also demonstrated the biological activity of the latter with an example of inhibition of influenza A virus replication in cell culture.

## Results and Discussion

Si–NH_2_ nanoparticles were synthesized by the hydrolysis of aminopropyltriethoxysilane (APTES). The resulting product is a clear aqueous solution containing nanoparticles in the form of low molecular weight polymer chains consisting of the [–Si(OH)(C_3_H_6_NH_2_)O–] monomer units as shown below.


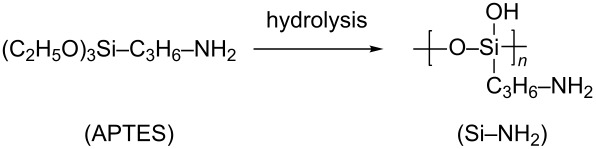


**Figure 1:**
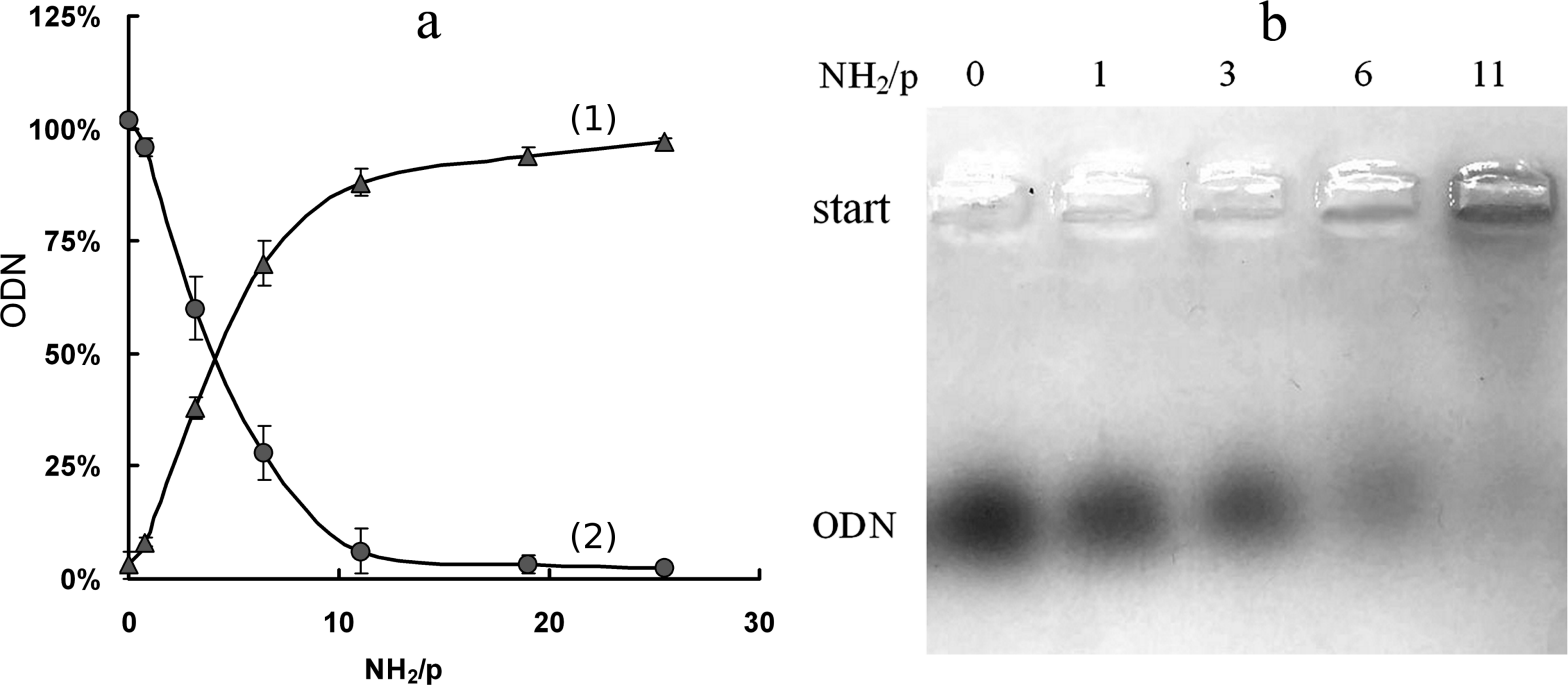
(a) Evaluation of molar ratio of the amino groups in the nanoparticles to the phosphate groups, NH_2_/p, for the complete binding of ODN(1) with Si–NH_2_ nanoparticles using the “acetone precipitation” technique. The amount of (1) bound and (2) unbound ODN(1) is shown. (b) Electrophoresis in agarose gel.

Due to the positively charged amino groups in Si–NH_2_ at the neutral pH values, the nanoparticles can be electrostatically conjugated with oligodeoxyribonucleotides (ODN) through the binding to the negatively charged internucleotide phosphate groups to form Si–NH_2_·ODN complexes. Like Si–NH_2_, the Si–NH_2_·ODN nanocomplexes are dissolved in aqueous solutions. The addition of NaCl did not lead to precipitation. Si–NH_2_ and Si–NH_2_·ODN can be precipitated by acetone. The unbound oligonucleotide after precipitation of Si–NH_2_·ODN remains in solution and can be detected spectrophotometrically. This “acetone precipitation” technique allowed us to evaluate the optimal molar ratio of the amino groups in the nanoparticles to the phosphate groups in an oligonucleotide (NH_2_/p ≈ 10) when preparing the Si–NH_2_·ODN nanocomplexes (Figure 1a).

A similar result was obtained when the mixture of ODN and Si–NH_2_ was analyzed by electrophoresis in agarose gel (Figure 1b). The higher the NH_2_/p ratio, the higher complexation occurred; at the ratio of 11, the band corresponding to the unbound oligonucleotide almost disappeared and the band corresponding to the Si–NH_2_·ODN complex could be observed at the top of the electrophoregram. The results indicate the binding of several Si–NH_2_ nanoparticles to one oligonucleotide molecule.

The binding of the Si–NH_2_ molecules to the internucleotide phosphate groups of an oligonucleotide leads to the formation of an ODN salt. In this work, this salt complex is conventionally named the Si–NH_2_·ODN nanocomplex.

The Si–NH_2_ nanoparticles and Si–NH_2_·ODN nanocomplexes were characterized by physico-chemical methods.

**Figure 2:**
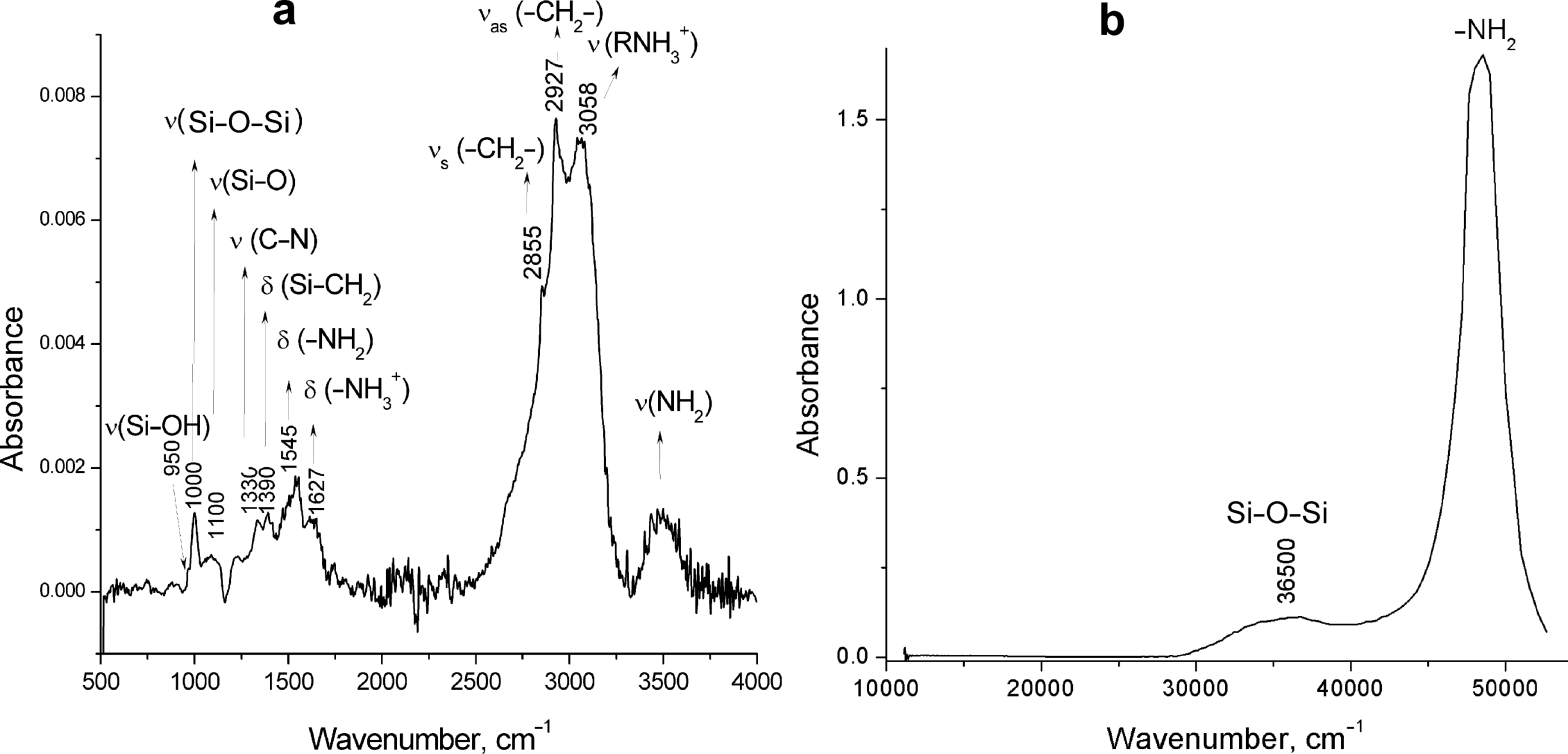
IR (a) and UV (b) spectra of Si–NH_2_.

The IR spectrum of Si–NH_2_ shows the local environment of the silicon atom (Figure 2a). The presence of the absorption bands that correspond to valence and deformation vibrations of the CH_2_, NH_2_, NH_3_^+^, Si–CH_2_, Si–OH, (Si–O)*_n_*, C–N groups [19] confirm the proposed structure of the Si–NH_2_ molecule. The splitting of the absorption band at 1085 cm^−1^, which corresponds to asymmetric valence vibrations of the Si–O bond, into two bands at 1100 cm^−1^ and 1000 cm^−1^ is typical for polymer structures with Si–O–Si fragments and indicates the presence of at least dimers in the Si–NH_2_ nanoparticles. The bands at 3058 cm^−1^ and 1627 cm^−1^ show the presence of the protonated NH_3_^+^ groups.

The electron absorption spectrum of the Si–NH_2_ solution has two bands at 49000 cm^−1^ and 36500 cm^−1^ (205 nm and 275 nm, respectively) (Figure 2b), the first of which corresponds to the (n–π*) transfer and belongs to the NH_2_ group [19]. The band at 36500 cm^−1^ can be attributed to the Si–O–Si structures because the absorption in the range of 35000–37000 cm^−1^ is characteristic for SiO_2_ nanoparticles and can be caused by electronic-type paramagnetic defects or n–π* transitions of the adsorbed organic ligand [19].

**Figure 3:**
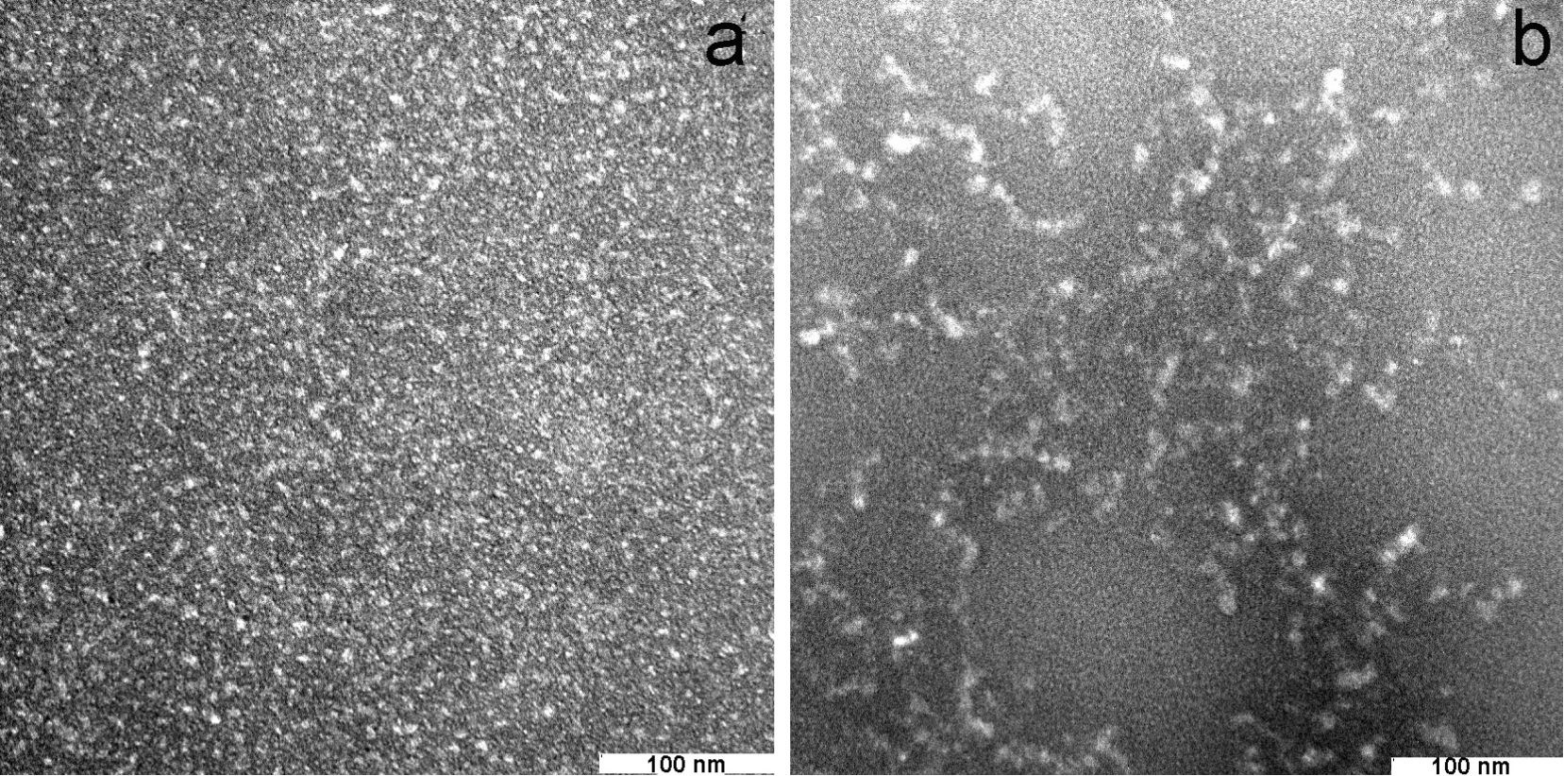
TEM images of Si–NH_2_ (a) nanoparticles and Si–NH_2_·ODN(1) nanocomplexes (b).

According to the transmission electron microscopy (TEM) images, there is a difference in morphology between Si–NH_2_ nanoparticles and their complexes with ODN. Discrete nanometer-sized nanoparticles (Si–NH_2_) are quite homogeneous (Figure 3a). Si–NH_2_·ODN(1) forms chain-like structures (Figure 3b), which can be explained by the formation of aggregates containing more Si–NH_2_ particles. Probably, the Si–NH_2_ counter ions in the Si–NH_2_·ODN complex are polymerized during the sample preparation (drying) due to their close proximity to one other.

The Si/P weight ratio for the analyzed Si–NH_2_·ODN(1) sample was evaluated by inductively coupled plasma mass spectrometry (ICP-MS). The experimental Si/P value (10.9) showed a reasonable correlation with the calculated data (10.0).

**Figure 4:**
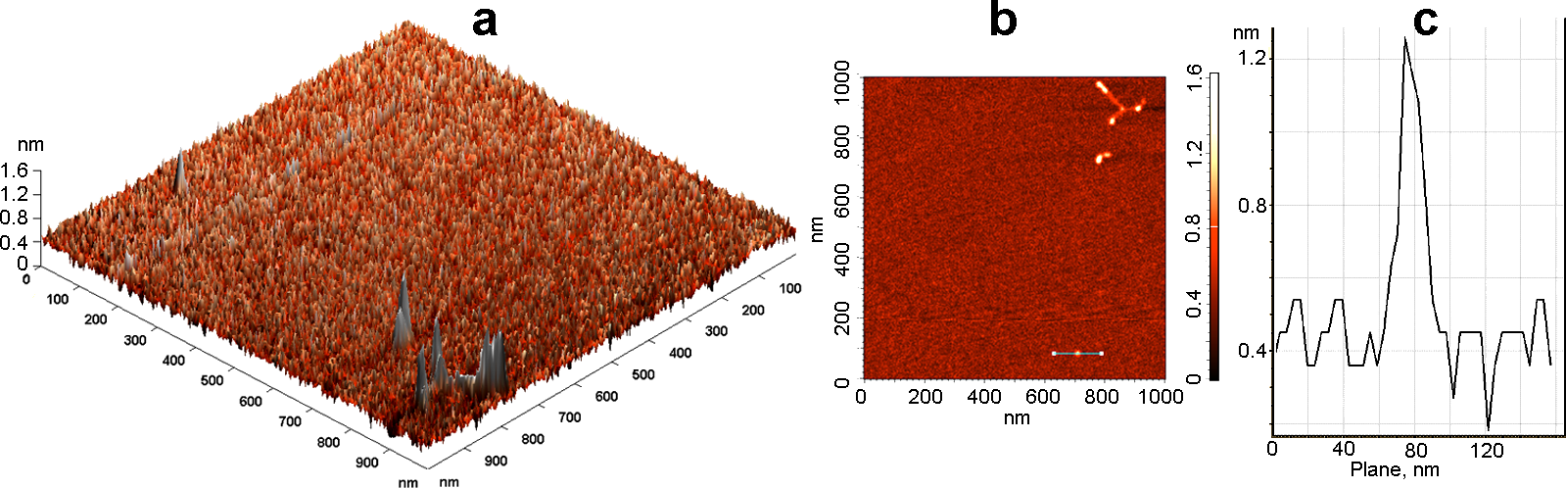
(a) 3D and (b) 2D AFM images of the Si–NH_2_·ODN(1) nanocomplex and (c) a suface profile along the line specified in (b).

The atomic force microscopy (AFM) image of the Si–NH_2_ nanoparticles was not obtained in a satisfactory quality because of the very small size of the particles. Fortunately, several slightly larger Si–NH_2_·ODN(1) nanocomplexes could be visualized (Figure 4). According to the AFM data, the size (height) of these nanocomplexes is 1.2–1.5 nm. As in the case of the TEM image (Figure 3b), the particles in the sample can form chain-like structures, which can increase the particle size detected by AFM.

Reasonable results on the diameter of the studied particles were obtained by dynamic light scattering (Table 1 and Table 2), which allows measurement of the particle size in solution (without effects of the drying process, which most likely leads to polymerization and agglomeration of the particles). The zeta potential of the studied samples was evaluated by phase analysis light scattering. The value of zeta potential of Si–NH_2_ is positive due to positively charged amino groups in neutral medium. When the nanoparticles were bound to an oligonucleotide, the zeta potential value, as expected, became negative (Table 1).

**Table 1:** Hydrodynamic diameter and zeta potential values of Si–NH_2_ and Si–NH_2_·ODN.

Sample	*d*, nm	Zeta potential, mV

Si–NH_2_	1.15 ± 0.17	+7.9 ± 0.6
Si–NH_2_·ODN(1)	2.70 ± 0.23	−14.3 ± 0.9
Si–NH_2_·ODN(2)	3.56 ± 0.37	
Si–NH_2_·ODN(3)	3.57 ± 0.34	
Si–NH_2_·ODN(2)/ODN(3)	4.82 ± 0.11	

**Table 2:** Diameter of Si–NH_2_ and Si–NH_2_·ODN complexes at different times after preparation.

Sample	Si–NH_2_						Si–NH_2_·ODN(1)					
*T* °C	4 °C			25 °C			4 °C			25 °C		

Days after preparation	1	30	90	1	30	90	1	30	90	1	30	90
*d*, nm^a^	1.05	1.21	1.10	1.16	1.23	–	2.65	2.82	2.76	2.45	2.78	–

^a^Standard deviation for all was between 0.12–0.27.

A rough estimation of the [–Si(OH)(C_3_H_6_NH_2_)O–] monomer volume based on the chemical bond lengths (≈0.4 nm^3^) allowed us to assume that the Si–NH_2_ nanoparticle (≈0.8 nm^3^) contains about two monomer units, i.e., dimers. The hydrodynamic diameter of the Si–NH_2_·ODN nanocomplex depends on the length of ODN: compare the sizes of Si–NH_2_·ODN(1) and Si–NH_2_·ODN(2) or Si–NH_2_·ODN(3) containing 15- and 19-meric oligonucleotides, respectively (Table 1).

The very small size of the Si–NH_2_ particles and Si–NH_2_·ODN complexes (1–5 nm) allows for the formation of true solutions. The prepared nanoparticles and nanocomplexes, Si–NH_2_ and Si–NH_2_·ODN, can be stored for a long time (at least for a month at 25 °C and three months at 4 °C) without changes in size (Table 2), which indicates no aggregation of these preparations occurred in these time frames.

Oligonucleotides in the Si–NH_2_·ODN nanocomplexes were shown to retain their ability to form complementary duplexes. The Si–NH_2_·ODN(2) and Si–NH_2_·ODN(3) nanocomplexes containing the complementary oligonucleotides have the same size (*d* ≈ 3.6 nm, Table 1); however, we observed an increase in the diameter to 4.8 nm for the particles in the mixture of these nanocomplexes. This fact indicates the formation of the complementary duplex. A similar increase in the size was observed when ODN(2) was added to the preformed Si–NH_2_·ODN(3) nanocomplex (Table 1), whereas the additional portion of ODN(3) did not lead to an increase in the size. The melting temperature values for the ODN(2)/ODN(3) duplex in the melting buffer (PBS) in the absence and in the presence of Si–NH_2_ nanoparticles (*T*_m_ = 57.5 ± 0.1 °C and 57.5 ± 0.3 °C, respectively) are equal, i.e., the presence of the nanoparticles does not prevent the duplex formation. At the same time, when we studied the duplex formation in the absence of PBS, the duplex was not formed without Si–NH_2_ (as expected), whereas the presence of nanoparticles provided the formation of the duplex with the *T*_m_ values of 37.6 ± 0.3 °C and 52.1 ± 0.1 °C at ≈1 mM and 10 mM concentration of Si–NH_2_, respectively. This experiment additionally confirms the role of Si–NH_2_ nanoparticles as counter ions when forming the Si–NH_2_·ODN complexes.

**Figure 5:**
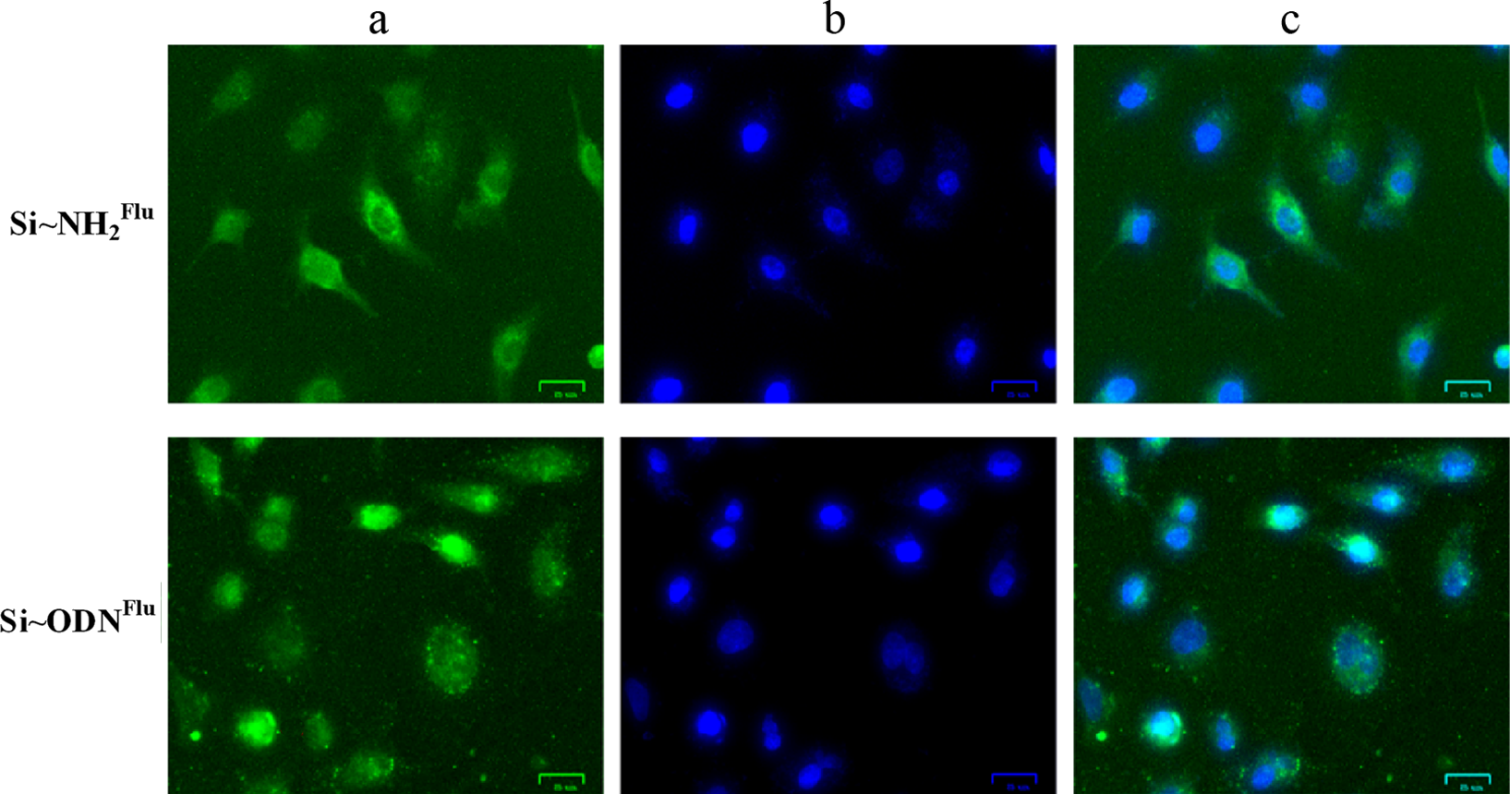
Fluorescence microscopy images of A549 human lung adenocarcinoma cells after their incubation with Si–NH_2_^Flu^ nanoparticles and Si–NH_2_·ODN(3)^Flu^ nanocomplexes. Fluorescein-labeled samples were detected in the green channel (488 nm) (a); DAPI-stained cell nuclei were detected in the blue channel (405 nm) (b); the superposition of all channels is shown in (c). The scale bar for all images corresponds to 25 μm.

Fluorescein-labeled nanoparticles (Si–NH_2_^Flu^) are readily detected by fluorescent microscopy in the A549 human epithelial cells after incubation for 8 h in the culture media (Figure 5, upper panel).

Si–NH_2_^Flu^ nanoparticles accumulate in microscopic fields related to the cytoplasm. Moreover, one can see that nuclei-related fields are less deeply colored. Under the same conditions, fluorescein-labeled oligonucleotide delivered into cells in the Si–NH_2_·ODN(3)^Flu^ nanocomplexes is detected not only in the cytoplasm, but also in nuclei of the human cells (Figure 5, lower panel). Some nuclei are colored more intensely than the cytoplasm in this case.

The biological properties of the proposed Si–NH_2_·ODN nanocomplexes are demonstrated with an example of the inhibition of the influenza A virus (IAV) replication in the cell culture.

The concentration of Si–NH_2_ and Si–NH_2_·ODN resulting in 50% MDCK cell death (TC_50_) was found to be 10–20 mM (for Si). The nontoxic concentration of the samples (0.014 mM for silicon) was used to study their ability to interact with the RNA target in cells with an example using the inhibition of influenza A virus (IAV) reproduction.

**Figure 6:**
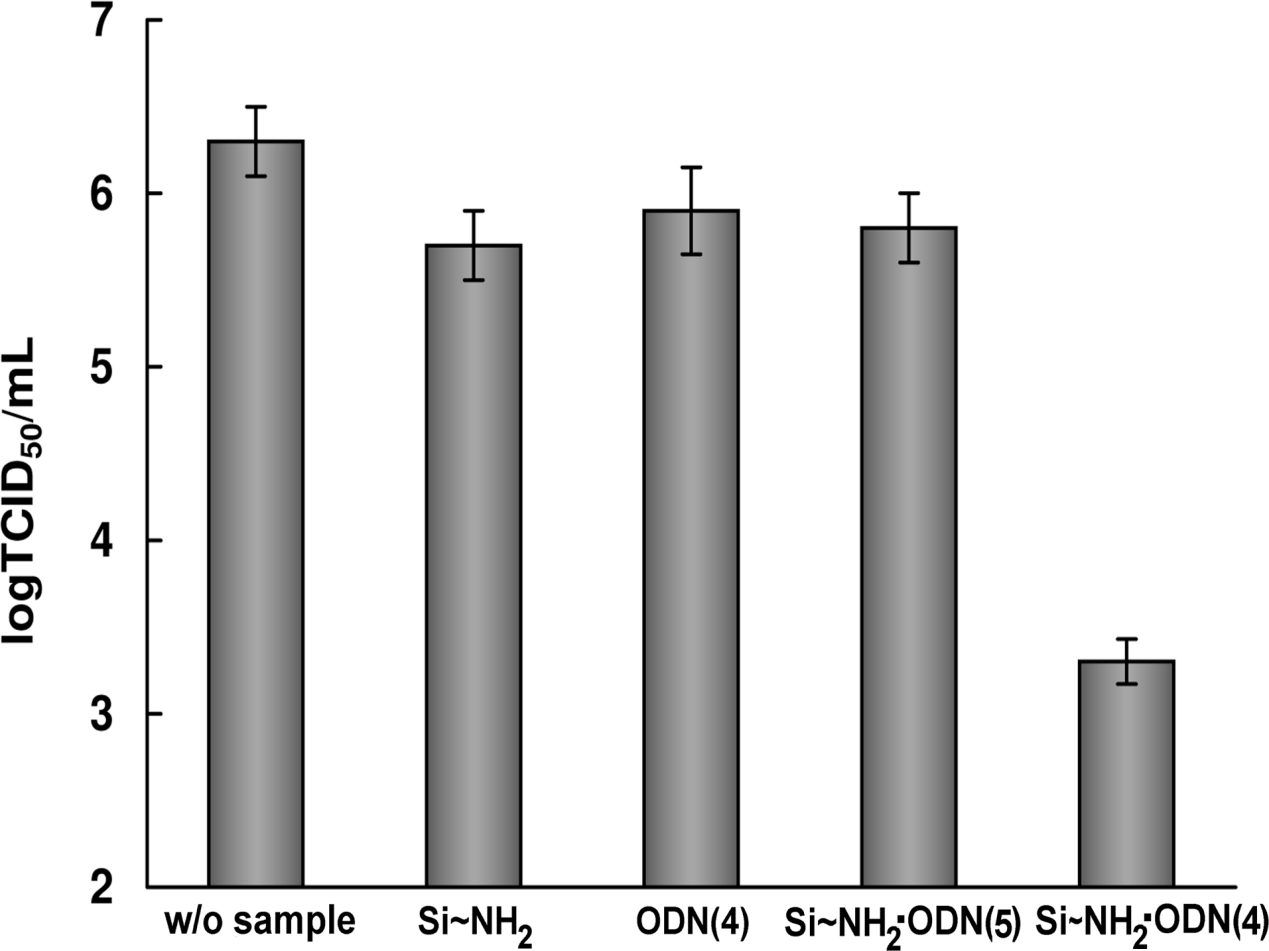
Virus titer (log TCID_50_/mL) of A/chicken/Kurgan/05/2005 virus (H5N1) in the presence of the samples from this study. Experiments were performed in three independent series.

The Si–NH_2_·ODN nanocomplex containing ODN(4) targeted to the 3’-noncoding regions of viral (−)RNA of IAV segment 5 and the control ODN(5) with a random sequence were assayed for the antiviral activity against avian influenza A virus H5N1. MDCK cells were infected at a multicipity of infection (MOI) of 0.1 TCID_50_/cell. The nanocomplex containing oligonucleotide ODN(4) complementary to viral RNA inhibited the virus reproduction by about three orders of magnitude (Figure 6), whereas Si–NH_2_ nanoparticles, unbound ODN(4), and the nanocomplex containing random ODN(5) was much less active.

The antiviral activity of the Si–NH_2_·ODN(4) nanocomplex was not inferior to that of the TiO_2_·PL–ODN nanocomplexes reported in our previous papers [20–22]. The high inhibition efficiency of the virus reproduction with the use of the Si–NH_2_·ODN nanocomplex is provided by the fact that the nanocomplex delivers ODN into the cytoplasm of the cell, and ODN can penetrate into the cell nuclei (Figure 5). The delivered ODN can interact with virus RNA via the antisense mechanism.

## Conclusion

We developed a very convenient and simple method for the preparation of non-agglomerated Si–NH_2_ nanoparticles and Si–NH_2_·ODN nanocomplexes. The Si–NH_2_ nanoparticles were obtained by a one-step synthesis from APTES, and the resulting product could be used without further purification to form Si–NH_2_·ODN nanocomplexes. The Si–NH_2_ nanoparticles and Si–NH_2_·ODN nanocomplexes were characterized by physico-chemical methods. The proposed nanocomplexes appeared to form very small nanoparticles (which are dissolved in aqueous solutions), then penetrate into the cellular cytoplasm to deliver oligonucleotides to the nucleus. The main advantage of the proposed Si–NH_2_ nanoparticles and Si–NH_2_·ODN nanocomplexes is that they are not prone to aggregation and form true aqueous solutions. The Si–NH_2_·ODN nanocomplexes exhibit biological activity, which has been shown with an example of the inhibition of influenza A virus replication. The proposed nanocomplexes can effectively and selectively interact with RNA targets in the cell culture.

## Experimental

### Materials and methods

All chemicals were obtained from commercial suppliers as follows: aminiopropyltriethoxysilane (APTES) and fluoresceinisothiocyanate (FITC), trypsin, penicillin-streptomycin, and L-glutamine (Sigma-Aldrich, USA); RPMI-1640 medium; antibiotics (BioloT, Russia); fetal calf serum (Gibco, USA). Сhicken erythrocytes, MDCK cells, and influenza A virus strain A/chicken/Kurgan/05/2005 (H5N1) were from FBRI Vector, Russia. Trypsin (1 mg/mL) and penicillin-streptomycin (100 U/mL) were stored at −80 °C. A549 human lung adenocarcinoma cells were from the Russian cell culture collection (Russian Branch of the ETCS, St. Petersburg, Russia). Oligodeoxyribonucleotides (Table 3) were synthesized by the phosphoramidite method on an ASM-800 DNA synthesizer (Biosset, Russia) using phosphoramidite monomers (Glen Research, USA).

**Table 3:** Oligodeoxyribonucleotides used in the work.

Oligonucleotide (5’→3’)	

AGCCGTACCCGCGCCp	ODN(1)
CTCCGAAGAAATAAGATCCp	ODN(2)
GGATCTTATTTCTTCGGAGp	ODN(3)
GGATCTTATTTCTTCGGAGp-Flu	ODN(3)^Flu^
GCAAAAGCAGGGTAGATAATCp	ODN(4)
GATCAACTCCATATGCCATGTp	ODN(5)

The optical absorption of oligonucleotides and their derivatives was measured on a Shimadzu U-1800 spectrophotometer (Shimadzu, Japan).

Inductively coupled plasma mass spectrometry (ICP-MS) was performed using an Agilent 7700 ICP MS (USA) spectrometer.

The IR spectrum of the Si–NH_2_ solution was recorded on a Cary 660 FTIR spectrometer (Agilent Technologies, USA) within the range of 4000–500 cm^−1^ at 4 cm^−1^ resolution, and 100 scans were accumulated in the attenuated total reflectance (ATR) mode using a GladiATR unit (Pike Technologies). The spectrum of the substance is presented as absorbance after computer processing, in particular, subtraction of the water spectrum (solvent) from the spectrum of the solution was performed (Figure 2a).

The electron spectrum of the Si–NH_2_ solution was taken on a Shimadzu UV-2501PC spectrophotometer (Shimadzu, Japan) in the absorbance mode. The spectrum was recorded with absorption compensation relative to water in the range 11000–54000 cm^−1^ in a quartz cuvette with an optical path length of 1 mm (Figure 2b).

The size and zeta potential values of Si–NH_2_ and Si–NH_2_·ODN were measured at 5–50 mM concentration (for Si) in physiological solutions or water on a Zetasizer Nano ZS Plus instrument (Malvern, UK) with the ratio of NH_2_/p = 10 in the Si–NH_2_·ODN nanocomplexes. The zeta potential values of the samples were obtained using a DTS1070 cuvette. Each sample was prepared at least in triplicate and measured three times at room temperature. The values of particle size and zeta potential were averaged over those experiments.

Transmission electron microscopy (TEM) images were taken on a JEM 1400 (Jeol, Japan) electron microscope at an accelerating voltage of 80 kV.

We analyzed a 35 mM solution of Si–NH_2_ nanoparticles and Si–NH_2_·ODN nanocomplexes with the ratio of NH_2_/p ≈ 10 in the latter case. The studied sample (10 μL) was mixed with a drop of 2% aqueous uranyl acetate; the mixture was covered with a standard carbon-coated copper mesh and after drying for one minute was fixed in a holder and introduced in the chamber of the electron microscope. The results are presented in Figure 3.

Atomic force microscopy (AFM) was performed on a Solver P47 Bio atomic force microscope (NT-МDT, Russia) in a tapping mode. The aqueous solution of the Si–NH_2_·ODN(1) sample (10 µL, 0.16 µM, NH_2_/p = 10) was applied to a freshly cleaved mica area of 25–30 mm^2^. The adsorption was held for one minute at room temperature; the remaining fluid was blown by air. The results are shown in Figure 4.

### Preparation of silicon–organic nanoparticles and Si–NH_2_·ODN nanocomplexes

APTES (8 mL, 34.16 mmol) was added dropwise to 100 mL of hot (70 °C) water. The mixture was stirred at this temperature for 15 h and then cooled to the room temperature. The concentration of the product (0.46 M) was evaluated by inductively coupled plasma mass spectrometry (ICP-MS) on an Agilent 7700 ICP-MS spectrometer or by titration of the amino groups with hydrochloric acid. The yield of the product was 95–97%.

The resulting Si–NH_2_ nanoparticles were attached to oligodeoxyribonucleotides using the electrostatic interaction between the negatively charged internucleotide phosphate groups and positively charged amino groups in Si–NH_2_. The amount of Si–NH_2_ nanoparticles necessary for the complete binding of an oligonucleotide was evaluated in the following experiments. In experiment (a), different amounts of Si–NH_2_ (0, 1, 3, 5, 10, 16, and 22 µL of 0.046 M solution) were added to ODN(1) (2.7 µL of 10^−3^ M; ≈40 nmol of phosphate groups) in water in seven vials. The volume of the mixtures was adjusted to 100 µL with water in each vial. After vigorous stirring for 1 min, the mixture was left at room temperature for 30 min to facilitate complexation. The formed Si–NH_2_·ODN(1) complex was precipitated by acetone (precipitate 1). Supernatants from each vial were separated, and the unbound ODN in these supernatants were precipitated by the addition of 1 µL of 3 M LiClO_4_ (precipitate 2). The precipitates 1 and 2 were dried at 60 °C to remove the acetone and were dissolved in 1 M NaCl. The amount of the bound (precipitate 1) and unbound (precipitate 2) oligonucleotide was evaluated spectrophotometrically at 260 nm. The results are presented in Figure 1a. In experiment (b), ODN(1) and Si–NH_2_ were mixed (as in (a)) and the samples (1 µL) were loaded onto 0.7% agarose gel prepared by heating of agarose in Tris-glycine buffer. Electrophoresis was performed at 5 V·cm^−1^ for 30 min. Oligonucleotide spots were visualized by StainsAll (Figure 1b).

In our experiments, the Si–NH_2_·ODN nanocomplexes were obtained by mixing 0.46 M Si–NH_2_ (2 µL) and ODN in 0.16 M NaCl or water (98 µL) while keeping the ratio between the amino and phosphate groups at NH_2_/p ≥ 10, unless otherwise specified.

### Formation of complementary duplexes

The Si–NH_2_·ODN(2) and Si–NH_2_·ODN(3) nanocomplexes were prepared by mixing 2 µL of 46 mM Si–NH_2_ (92 nmol) and 2 µL of 0.24 mM ODN(2) or ODN(3) (9.2 nmol of phosphate groups) in physiological solution (96 µL). The formed Si–NH_2_·ODN(2) and Si–NH_2_·ODN(3) were combined, and their sizes were measured before and after mixing. In another experiment, ODN(3) or ODN(2) (2 µL 0.24 mM) was added to the Si–NH_2_·ODN(2) nanocomplex prepared as above, and the sizes of the particles were measured.

### Thermal denaturation of ODN duplexes

Thermal denaturation of ODN duplexes was studied using a UV-1800 spectrophotometer equipped with a TMSPC-8 instrument (Shimadzu, Japan) for analyzing the melting temperature of the samples. The mixtures of ODN(2) and ODN(3) (10 μL, 30 μM, each) in a PBS buffer (80 μL), pH 7.4, or in water (80 μL) was annealed and gradually heated from 5 °C up to 90 °C and cooled from 90 °C to 5 °C (0.5 °C/min). The same mixtures in the presence of the Si–NH_2_ nanoparticles (2.6 μL, 0.042 M or 0.42 M, which correspond to a NH_2_/p ratio of 10 or 100, respectively) were subjected to the same procedure.

### Preparation of fluorescently labeled nanoparticles (Si–NH_2_^Flu^) and nanocomplexes (Si–NH_2_·ODN^Flu^)

A mixture of 0.46 M Si–NH_2_ (5 µL, 2.3 µmol) and 2 mM FITC (45 µL, 0.09 µmol) was kept for 1 h at 60 °C. The reaction mixture was loaded onto a column (100 µL) with LichroPrep-C18 pre-equilibrated with 0.05 M LiClO_4_. Chromatography was carried out in manual mode by stepwise elution using solutions of acetonitrile in water containing 0.05 M LiClO_4_ (0, 5, 10, 20, and 50%). Fractions (by 100 µL) were evaporated and analyzed by TLC in an i-propanol-NH_4_OH-water mixture (6:1:3) on the TLC plates (Merck, Germany). The product containing the fluorescein residue (Si–NH-Flu) was revealed mainly in the second fraction (5% CH_3_CN). The mobility of the product in TLC (*R*_f_ ≈ 0.5) differed from that of hydrolyzed FITC (*R*_f_ ≈ 0.65). The concentration of Si–NH-Flu (33 µM) was evaluated spectrophotometrically using the molar absorption coefficient for fluorescein (ε_495_ = 74000 cm^−1^·M^−1^).

For fluorescence microscopy, the Si–NH-Flu product (3 µL, 0.1 nmol of the Flu residue) and 46 mM Si–NH_2_ (1 µL, 46 nmol) were mixed in 46 µL of water, resulting in the solution of Si–NH_2_^Flu^ (≈1 mM for Si and 2 µM for Flu). The labeled Si–NH_2_·ODN(3)^Flu^ nanocomplex of the same concentration for Si and Flu was prepared by mixing 46 mM Si–NH_2_ (1 µL, 46 nmol) with 10^−4^ M ODN(3)^Flu^ (1 µL, 0.1 nmol) in 48 µL of water.

### Penetration of nanocomplexes into eukaryotic cells

A549 human lung adenocarcinoma cells were cultivated in Iscove’s modified Dulbecco’s media (IMDM; Sigma-Aldrich) with 10% FBS (Gibco BRL Co., USA), 2 mM L-glutamine (Sigma-Aldrich), and 100 U/mL penicillin/streptomycin (Gibco BRL Co., USA). The cells were seeded at 2 × 10^5^ cells/chamber in 4-well microscopic chambered cell culture slides (Fisher Scientific, USA) and cultivated for 24 h in a humidified incubator at 37 °C, 5% CO_2_. The fluorescein-labeled Si–NH_2_^Flu^ nanoparticles and Si–NH_2_·ODN(3)^Flu^ nanocomplex (final concentration in medium 0.1 mM for Si and 0.2 µM for the Flu residue) were added to the culture media. After incubation for 8 h at 37 °C, the medium was removed, and the cells were rinsed twice with phosphate buffer saline (PBS), followed by fixation with 4% formaldehyde (w/v) in PBS for 20 min at room temperature. The glass slides were washed twice with PBS and mounted in ProLong antifade mountants (ThermoFisher Scientific, USA). The cells were analyzed with a ZOE Fluorescent Cell Imager (Bio-Rad, USA). Laser lines at 405 nm (for nuclei, DAPI blue) and 488 nm (for fluorescein-labeled samples, green) were used. The results are shown in Figure 5.

### Cell viability assays

The experiments were carried out similarly as described in [20]. In short: Two days after the formation of a continuous monolayer of MDCK cells at 37 °C and 5% CO_2_, the cells were washed with nutrient medium without serum. The Si–NH_2_ or Si–NH_2_·ODN samples were diluted with RPMI-1640 medium to the needed concentration (2–35 mM for Si) and incubated with MDCK cells at 37 °C and 5% CO_2_ for two days. Destructive changes in the cells were evaluated by an inverted microscope. MDCK cells without studied samples were used as a control and taken for 100%. The cells were stained with trypan blue [23], and the number of viable cells were counted in a Goryaev chamber.

### Antiviral activity of nanocomplexes

The antiviral activity experiments were performed analogously as described in [22]. The influenza A virus (IAV) strains and MDCK cells were prepared as in [22]. The cells at ≈80% confluence were initially infected with A/chicken/Kurgan/05/2005 virus (H5N1), which was added in each well in RPMI-1640 medium (100 μL) containing trypsin (2 μg/mL) at a MOI of 0.1. The control sample was RPMI-1640 medium (100 μL) containing trypsin (2 μg/mL). After 1 h of virus adsorption at room temperature, the virus-containing medium was removed, and the cells were rinsed with RPMI-1640 medium without trypsin. The Si–NH_2_, ODN(4), Si–NH_2_·ODN(4), and Si–NH_2_·ODN(5) studied samples taken in RPMI-1640 medium without trypsin (100 μL/well) at a concentration of 0.014 mM for Si and 0.1 μM for ODN were applied to the infected MDCK cells, followed by incubation for 4 h at 37 °C, 5% CO_2_, and 100% humidity. After incubation for 4 h at room temperature, the medium containing the sample was removed, the cells were rinsed with RPMI-1640 medium without trypsin, and the same medium containing trypsin was added in each well (100 μL). After incubation for 48 h, serial ten-fold dilutions (from 10^–1^ to 10^–8^) of the cultural virus-containing liquid from each well were applied to MDCK cells for 48 h to evaluate the virus titer. The presence of the virus was visually determined under a microscope by the cytopathic action and in the hemagglutination reaction with a 1% suspension of chicken erythrocytes. The virus titer was expressed in terms of log TCID_50_/mL (Figure 6). The titer was evaluated by noting the highest dilution of the virus, which caused the hemagglutination reaction.

## References

[1] Levina A, Ismagilov Z, Repkova M, Shatskaya N, Shikina N, Tusikov F, Zarytova V (2012). J Nanosci Nanotechnol.

[2] Levina A S, Ismagilov Z R, Repkova M N, Shikina N V, Baiborodin S I, Shatskaya N V, Zagrebelnyi S N, Zarytova V F (2013). Russ J Bioorg Chem.

[3] Liu Y, Lou C, Yang H, Shi M, Miyoshi H (2011). Curr Cancer Drug Targets.

[4] Roy I, Stachowiak M K, Bergey E J (2008). Nanomedicine (N Y, NY, U S).

[5] Roy I, Ohulchanskyy T Y, Bharali D J, Pudavar H E, Mistretta R A, Kaur N, Prasad P N (2005). Proc Natl Acad Sci U S A.

[6] Cheang T Y, Tang B, Xu A W, Chang G Q, Hu Z J, He W L, Xing Z H, Xu J B, Wang M, Wang S M (2012). Int J Nanomed.

[7] Neumeyer A, Bukowski M, Veith M, Lehr C-M, Daum N (2011). Nanomedicine (N Y, NY, U S).

[8] Kneuer C, Sameti M, Haltner E G, Schiestel T, Schirra H, Schmidt H, Lehr C-M (2000). Int J Pharm.

[9] Park J-H, Gu L, von Maltzahn G, Ruoslahti E, Bhatia S N, Sailor M J (2009). Nat Mater.

[10] Kreuter J (2001). Adv Drug Delivery Rev.

[11] Mahajan S D, Law W C, Aalinkeel R, Reynolds J L, Nair B B, Sykes D E, Yong K T, Roy I, Prasad P N, Schwartz S A (2012). Int J Nanomed.

[12] Wang K, He X, Yang X, Shi H (2013). Acc Chem Res.

[13] Lee J E, Lee N, Kim T, Kim J, Hyeon T (2011). Acc Chem Res.

[14] Tang F, Li L, Chen D (2012). Adv Mater.

[15] Chen G, Roy I, Yang C, Prasad P N (2016). Chem Rev.

[16] Heidegger S, Gößl D, Schmidt A, Niedermayer S, Argyo C, Endres S, Bein T, Bourquin C (2016). Nanoscale.

[17] Liberman A, Mendez N, Trogler W C, Kummel A C (2014). Surf Sci Rep.

[18] Kumar R, Roy I, Ohulchanskyy T Y, Goswami L N, Bonoiu A C, Bergey E J, Tramposch K M, Maitra A, Prasad P N (2008). ACS Nano.

[19] Pan H, Li G-L, Liu R-Q, Wang S-X, Wang X-D (2017). Appl Surf Sci.

[20] Levina A S, Repkova M N, Ismagilov Z R, Shikina N V, Malygin E G, Mazurkova N A, Zinov'ev V V, Evdokimov A A, Baiborodin S I, Zarytova V F (2012). Sci Rep.

[21] Levina A S, Repkova M N, Mazurkova N A, Makarevich E V, Ismagilov Z R, Zarytova V F (2015). Int J Antimicrob Agents.

[22] Levina A S, Repkova M N, Bessudnova E V, Filippova E I, Mazurkova N A, Zarytova V F (2016). Beilstein J Nanotechnol.

[23] Altman S A, Randers L, Rao G (1993). Biotechnol Prog.

